# The Author's Method

**Published:** 1840-07

**Authors:** 


The following passage from Mr. Koecker's work, entitled "Principles of Dental Surgery" published in London, A. D. 1826, is presented to our readers for the purpose of eliciting from some of our correspondents, an article on the same subject, invalidating or confirming the doctrines therein advanced. The subject is an important one, and the younger members of the profession will, no doubt, be thankful for all the light that experience or observation can supply.

### THE AUTHOR'S METHOD

*Of treating the Lining Membrane of the Tooth when exposed.*

I require for this the following apparatus: 1. A small iron wire, fastened to an ivory handle. The extremity of this wire I file to the size of the exposed surface of the nerve, and bend the wire in such a direction as to enable me to touch the exposed part of the membrane, without touching any other part of the tooth or the mouth. 2. A thick tallow candle, with a large wick.

I direct the patient to discharge all the saliva he may have in his mouth, and then to incline his head backwards against the head support of my operating chair. I put the candle into his left hand, and direct him to hold it in such a position that the flame of it may be on a level with his mouth, and about eight inches from it; I now place myself on the right side of the patient, and, holding his lips sufficiently open with my left hand, to prevent the instrument from touching them, I again dry the cavity as perfectly as possible with a lock of cotton fastended to the point of the cauterizing wire. Having effected this, I throw away the cotton from the extremity of the wire, and make it red hot in the flame of the candle. With the wire thus heated, I touch the exposed part very rapidly, so that its surface contracts, without, however, suffering it to penetrate deeply into the nerve, or to touch any part of the bony structure; as this would inevitably bring on suppuration and destruction of the whole lining membrane. The bleeding spot must be touched very quickly with the hot wire, which is sometimes necessary to be repeated two or three times before the parts are sufficiently contracted. The wire should be perfectly red hot, for in this state the cautery acts suddenly, and almost entirely without pain; but when heated to any temperature short of that of red heat, much pain and inflammation are generally produced. This operation is, indeed, so slightly painful, that I have been solicited by my patients to repeat it, although they had required much persuasion to induce them in the first instance to suffer its application. It, however, must be performed very adroitly, and without any loss of time. To prevent the flow of saliva to interfere, the patient must be desired not to close his lips, but to keep his mouth wide open, until the whole of the operation is finished, which he is capable to do for a certain time only.

When the hæmorrhage has been arrested in this way, and an artificial cicatrix formed, I wash the cavity, as before the cauterization, with warm water. I carefully remove every particle of the ashes or matter that may have been left by the cauterization, taking great care not to wound the membrane again.

The nerve, which, before cauterization, had a fleshy appearance, is, after this operation, like a black point. I take care not to disturb this point, for if the black scar be removed, a new wound will be formed and bleeding again will ensue; but I leave the future healing altogether to nature, and only caution my patient against using such things as might interfere with its salutary operations.

Having thus far removed all possible cause of future disease and irritation, in order to prevent any unnecessary exposure of the nerve, by which inflammation and destruction of it might be produced, I now terminate the operation by fulfilling the third indication, that is, to protect the nerve against injurious impressions from without, by filling up the cavity of the tooth with metal. Having again perfectly dried the cavity, I now take a small pl te of very thin lead leaf, and lay it upon the exposed nerve, and on the immediately surrounding bony parts. I next carefully fill up the whole cavity with gold. The dressing of the cavity and the firm insertion of the two different metals also, must be completed before the patient can be allowed to close his mouth, which is not unfrequently very difficult.

